# Photographic Illustrations of Skin Diseases

**Published:** 1889-12

**Authors:** 


PHOTOGRAPHIC ILLUSTRATIONS OF SKIN DISEASES. By Geo. H. Fox, A. M., M. D. Parts 9 and 10. Price, $2.00 each. E. B. Treat, 771 Broadway, N. Y.

Of the accuracy of these illustrations and of the clearness of the accompanying text, we have frequently written. We need only add here that Parts 9 and 10 fully bear out the excellent character of Professor Fox's work.

